# Role of Specificity Protein 1 (SP1) in Cardiovascular Diseases: Pathological Mechanisms and Therapeutic Potentials

**DOI:** 10.3390/biom14070807

**Published:** 2024-07-07

**Authors:** Jie Ding, Aminah I. Fayyaz, Yuchuan Ding, Dandan Liang, Ming Luo

**Affiliations:** 1 School of Medicine, Tongji University, Shanghai 200092, China; djtongji0504@tongji.edu.cn (J.D.); dandanliang@tongji.edu.cn (D.L.); 2 State Key Laboratory of Cardiovascular Diseases, Shanghai East Hospital, School of Medicine, Tongji University, Shanghai 200120, China; 3 Shanghai Arrhythmia Research Center, Shanghai East Hospital, School of Medicine, Tongji University, Shanghai 200120, China; 4 Department of Neurosurgery, Wayne State University School of Medicine, Detroit, MI 48201, USA; aminah.fayyaz@wayne.edu (A.I.F.); yding@med.wayne.edu (Y.D.)

**Keywords:** SP1, cardiovascular disease, coronary heart disease, ischemia-reperfusion injury, cardiomyopathy, arrhythmia

## Abstract

In mammals, specificity protein 1 (SP1) was the first Cys2-His2 zinc finger transcription factor to be isolated within the specificity protein and Krüppel-like factor (Sp/KLF) gene family. SP1 regulates gene expression by binding to Guanine–Cytosine (GC)-rich sequences on promoter regions of target genes, affecting various cellular processes. Additionally, the activity of SP1 is markedly influenced by posttranslational modifications, such as phosphorylation, acetylation, glycosylation, and proteolysis. SP1 is implicated in the regulation of apoptosis, cell hypertrophy, inflammation, oxidative stress, lipid metabolism, plaque stabilization, endothelial dysfunction, fibrosis, calcification, and other pathological processes. These processes impact the onset and progression of numerous cardiovascular disorders, including coronary heart disease, ischemia-reperfusion injury, cardiomyopathy, arrhythmia, and vascular disease. SP1 emerges as a potential target for the prevention and therapeutic intervention of cardiac ailments. In this review, we delve into the biological functions, pathophysiological mechanisms, and potential clinical implications of SP1 in cardiac pathology to offer valuable insights into the regulatory functions of SP1 in heart diseases and unveil novel avenues for the prevention and treatment of cardiovascular conditions.

## 1. Introduction

Cardiac disorders exhibit high rates of illness and death and represent the primary global cause of mortality [1,2,3]. The pathogenesis and development of heart diseases are influenced by a group of transcription factors (TFs) operating at various temporal and spatial dimensions [4]. TFs, which possess DNA-binding domains conserved across different species, are proteins crucial in regulating gene expression [5,6]. Through interactions with regulatory sites, TFs form gene regulatory networks that steer development and physiology [7]. Their role is paramount in controlling gene expression by binding to promoters or enhancers in DNA sequences and modulating the transcription of target genes [8]. Disruption of gene regulation lies at the core of numerous human diseases, with mutations in TFs and TF binding sites obviously being the primary culprits [7]. However, our understanding of the involvement of TFs in cardiac diseases remains somewhat limited.

Among these TFs, specificity protein 1 (SP1) is the first transcription factor identified in mammals and the most active member within the Sp/KLF family [9,10]. SP1 has been implicated in various biological processes, especially tumorigenesis in several cancers, including breast, gastric, pancreatic, and lung [11,12]. Recent advances in both basic and clinical research have unveiled the additional roles of SP1 in the onset and progression of heart diseases. It plays a critical role in regulating myocardial apoptosis, myocardial fibrosis, inflammation, vascular calcification, and other pathological processes related to cardiovascular ailments [13,14]. Given its multifaceted involvement, SP1 holds promise for enhancing diagnostic and therapeutic strategies for cardiovascular diseases. Hence, this review provides an in-depth exploration of SP1’s structure, biological functions, and impact on cardiac pathology.

## 2. SP1 Gene and Protein Structure

**Figure 1:**
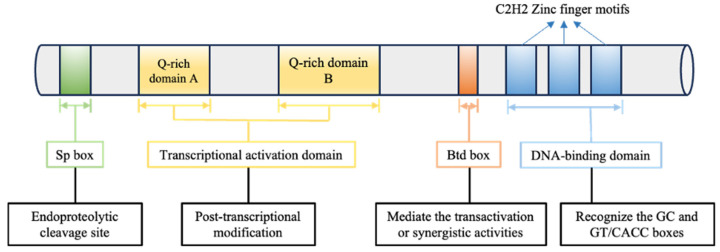
Structure of the SP1 gene. Different colors indicate various conserved domains, including the transcriptional activation domain, DNA-binding domain, Btd (buttonhead) box, and Sp box. The specific domain and unique biological functions are annotated.

The gene encoding SP1 is located on human chromosome 12q12–q13.2, with 12q13.1 being the most probable locus [15]. Key conserved domains within SP1 encompass the transcriptional activation domain, DNA-binding domain, buttonhead (Btd) box, and Sp box. These conserved elements dictate the structural and functional attributes of SP1 (Figure 1). The transcriptional activation domain, harbored at the N-terminus of SP1, comprises two glutamine-rich transactivation domains, A and B. These domains serve as hotspots for diverse post-transcriptional modifications. Near the C-terminus of SP1, the DNA-binding domain predominantly consists of three contiguous Cys2-His2 zinc finger motifs that recognize the GC and GT/CACC boxes, consequently governing the transcription of target genes. While the functions of the Btd box and Sp box remain relatively unexplored, the Btd box is thought to mediate transactivation or synergistic activities, and the Sp box contains an endoproteolytic cleavage site [9,16].

SP1 initially forms a tetramer that bends SP1-bound DNA to create a DNA loop. Tetramers consisting of identical subunits can assemble only as two types of ring structures. The first ring type, C4, exhibits quadruple rotational symmetry, while the second type, D2, demonstrates inversion of consecutive monomers (including zinc finger domains). The DNA loop is hypothesized to be more likely to adopt the D2 morphology. Meanwhile, the DNA loop facilitates the transfer of distantly located SP1 proteins to the proximal promoter region. Subsequently, SP1 assembles multiple tetramers stacked at the DNA loop junction, leading to robust activation of transcription [17].

## 3. Distribution and Biological Functions of SP1

SP1 is expressed in nearly all tissues, particularly in organs such as nerves, lungs, muscles, and the heart [18]. It is also highly increased in various tumors, including lung, gastric, breast, colorectal, and pancreatic cancers [11,19]. Additionally, SP1 exhibits prominent expression during the embryonic stages, with the expression level gradually declining as the fetus develops and ages [20]. The predominant cellular locations for SP1 expression are the nucleus and cytoplasm, where it is regulated by its glycosylation/phosphorylation state, as well as by insulin and other hormones [21].

SP1 plays a crucial role in regulating numerous biological processes, including DNA damage repair, chromatin remodeling, immune responses, cell proliferation, cell growth, and tumorigenesis [10,22,23,24]. In the cardiovascular area, multiple studies have illustrated the role of SP1 in gene transcription, including genes like insulin-like growth factor 1 (IGF-1), fibroblast growth factor 2 (Fgf2), cardiac troponin I (cTnI), and vascular endothelial growth factor (VEGF) in cardiomyocytes [25,26,27,28]. By binding to Guanine–Cytosine (GC)-rich sequences on target gene promoters and/or undergoing various post-translational modifications such as phosphorylation, acetylation, glycosylation, and proteolysis, SP1 either promotes or inhibits gene transcription and modulates the activity of encoded proteins and the expression of non-coding genes [29]. Consequently, SP1 influences the development of cardiac diseases by participating in pathological processes like inflammation, cell growth, angiogenesis, and fibrosis.

## 4. SP1 in Cardiac Diseases

### 4.1. Myocardial Ischemia-Reperfusion Injury

Myocardial ischemia-reperfusion injury (MIRI) represents a common challenge in the clinical management of coronary atherosclerotic heart disease. The pathogenic mechanisms of MIRI primarily encompass calcium overload, oxidative stress induced by oxygen-free radicals, and disruptions in energy metabolism [30]. Recent research indicates that SP1 contributes to the progression of MIRI through its impact on apoptosis, inflammatory responses, and oxidative stress-induced pathways.

Calcium overload is intricately linked to mitochondrial dysfunction and membrane permeability. MIRI induces calcium overload by causing an excessive buildup of Ca(2+) inside the mitochondria, leading to mitochondrial dysfunction, myocardial cell injury, and death. The sarcoendoplasmic reticulum Ca(2+) ATPase 2a (SERCA2a) is crucial for calcium transport during excitation–contraction coupling and is essential for maintaining myocardial contractile function and cellular calcium levels [31]. A recent study has shown that down-regulation of SP1 reduces SERCA2a activity during MIRI, resulting in calcium overload in cardiomyocytes. Pre-treatment with luteolin, a flavonoid, can mitigate myocardial injury from calcium overload by enhancing SP1 binding to the SERCA2a promoter region, increasing its transcriptional activity, and providing myocardial protection [32]. Additionally, the mitochondrial calcium uniporter (MCU) is a selective calcium channel in the inner mitochondrial membrane, regulated by complex interactions with proteins like MICU1, MCUR1, EMRE, and MICU2 to control mitochondrial calcium levels. Researchers have demonstrated that miR-181c is upregulated during MIRI and alters mitochondrial calcium levels via the miR-181c↑→ mt-COX1↓→ ROS↑→ SP1↓→ MICU1↓ signaling pathway, leading to calcium overload and myocardial apoptosis [33]. Therefore, SP1 plays a critical role in calcium overload, and upregulating SP1 expression may alleviate MIRI by increasing MICU1 and SERCA2a levels and preventing apoptosis.

In the context of ischemia-reperfusion, reactive oxygen species (ROS) act on cell membranes to produce leukotrienes (LTs), which recruit and activate neutrophils, causing tissue damage. Upon reperfusion, activated neutrophils consume excess oxygen, generating oxygen-free radicals, which lead to tissue damage. Intercellular adhesion molecule-1 (ICAM-1) is a cell surface glycoprotein that promotes neutrophil adhesion and tissue damage during MIRI. Studies have revealed that SP1 binds to a signal transducer and activator of transcription 3a (Stat3a) to form a complex that binds to gamma-interferon activation site (GAS) sequences in the ICAM-1 promoter, enhancing ICAM-1 transcription and exacerbating myocardial injury. Conversely, inhibiting SP1 and Stat3a can reduce ICAM-1 transcription and alleviate myocardial damage [34]. However, regulation of SP1 on CD39 has a protective effect during MIRI. Hypoxia/ischemia-induced inflammation triggers vascular endothelial cells to release adenine nucleotides [35], requiring CD39-mediated conversion to adenosine to halt the inflammatory response. SP1 regulates CD39 transcription by binding to its promoter region, promoting adenosine production, and protecting cardiac tissue [36]. Therefore, SP1 may be involved in the regulation of the myocardial ischemia-reperfusion injury (MIRI) inflammatory response. Further studies are necessary to elucidate the regulatory role of SP1, which could potentially emerge as a novel target for managing the MIRI inflammatory response.

More oxygen-free radicals are created during myocardial reperfusion when compared to the amount produced by ischemia, inducing autophagy, ischemic cardiomyocyte death, and cardiac dysfunction (31). Autophagy is the process of transporting damaged, denatured, or aged organelles to lysosomes for degradation. Previous studies have shown that Poly (ADP-ribose) polymerase 1 (PARP1) is a post-translational modification enzyme that attaches ADP-ribose polymer chains to target proteins and promotes DNA repair [37]. Xu et al. found that silencing SP1 protects cardiomyocytes from MIRI by inhibiting PARP1, mainly by suppressing cardiomyocyte autophagy [38]. In this paper, the authors have demonstrated that silencing SP1 has a protective effect on MIRI, which is inconsistent with the above study. Therefore, this may be due to the fact that the role of SP1 in different pathological processes of MIRI may vary. In addition, the impact of SP1 on MIRI development can also be achieved by regulating cardiomyocyte autophagy.

### 4.2. Coronary Atherosclerotic Heart Disease

Coronary atherosclerotic heart disease is characterized by myocardial ischemia, hypoxia, or necrosis resulting from vascular stenosis or obstruction caused by atherosclerotic lesions in the coronary arteries. Emerging data indicate the pivotal involvement of SP1 in key processes during atherosclerosis (AS) progression, such as inflammation, lipid metabolism, plaque stability, endothelial dysfunction, and fibrosis [39].

Macrophages are pivotal in the inflammatory response and foam cell formation during AS development. Exploring new targets that can modulate macrophage inflammatory responses may offer innovative approaches for AS prevention and treatment. Studies since 2007 have investigated the link between gene polymorphisms of proline-rich/serine-rich coiled-coil protein 1 (PSRC1) and AS progression. Previous research by Pan et al. demonstrated that PSRC1 over-expression in macrophages reduced inflammatory factor expression and secretion in AS models in ApoE^−/−^ mice. The interaction of PSRC1 with Annexin A2 (ANXA2) in macrophages can inhibit AS progression, and SP1 acts as an upstream transcription factor of PSRC1, influencing AS development through macrophage inflammatory responses [40].

Foam cell formation is triggered by excessive cholesterol accumulation in macrophages or vascular smooth muscle cells. Transporters like ATP-Binding Cassette Subfamily A Member 1 (ABCA1) and Scavenger Receptor Class B Member 1 (SR-BI) play crucial roles in cholesterol movement between cells and extracellular receptors. ABCA1 promotes cholesterol efflux in macrophages, with PI3K and PKCζ mediating SP1 phosphorylation to enhance ABCA1 expression [41,42]. Similarly, inhibiting the expression of SR-BI promotes lipid accumulation in foam cells, which is attributed to the reduction in SP1 binding to the SR-BI promoter, leading to decreased reverse cholesterol transport [43]. These findings suggest that SP1 could be a promising target for dyslipidemia treatment in AS patients by regulating ABCA1 and SR-BI.

Enhancing plaque stability is vital for preventing the severe consequences of AS. The intracellular enzyme P4Ha1 is essential for collagen maturation and secretion, thereby increasing AS plaque stability. SP1 has been identified as a key transcription factor of P4Ha1, with activation of the Akt signaling pathway promoting phosphorylation and subsequent transcriptional activity of SP1 and increasing P4Ha1 expression to enhance plaque stability in AS [44,45]. Vascular endothelium serves as a physiological barrier and sustains the normal function of blood vessels by regulating blood fluidity, fibrinolysis, vascular tone, and platelet aggregation. However, in specific pathological conditions like high glucose levels, circulating low-density lipoprotein, or accumulation of apolipoprotein B, the vascular endothelium enlists macrophages to engage in inflammatory responses that foster atherosclerotic (AS) plaque formation. The mechanisms of cell-to-cell communication in AS remain incomplete, but studies have shown that macrophages and endothelial cells are interconnected and expedite AS progression through exosomes. Recent research by Liu et al. revealed that macrophage-derived exosomes containing miR-4532 target SP1, leading to SP1 degradation by binding to the SP1 3′ untranslated region (3′ UTR). SP1 degradation activates downstream NF-κB P65, disrupting endothelial cell function and potentially advancing the pathological process of AS [46]. Furthermore, mounting evidence indicates that the occurrence of endothelial cell pyroptosis assumes a new role in the development of AS [47]. Pyroptosis is a type of pro-inflammatory programmed cell death distinguished by the activation of the Nod-like receptor family pyrin domain containing 3 (NLRP3), resulting in rapid membrane disintegration and the release of cellular components and pro-inflammatory agents. Research has revealed that the interaction between SP1 and the lncRNA Gaplinc triggers binding to the NLRP3 promoter, thereby activating its transcription. Consequently, this activation encourages endothelial cell pyroptosis and malfunctions [48]. Collectively, SP1 plays a crucial role in vascular endothelial function, and its activation could aid in the prevention and treatment of AS.

Moreover, SP1 has been implicated in regulating cardiac remodeling and myocardial fibrosis post-myocardial infarction (MI). During MI, irreversible cardiomyocyte loss occurs due to oxidative stress and inadequate oxygen and blood supply to the heart. Subsequent dead cell debris and toxic substances trigger left ventricular remodeling, culminating in heart failure. In cardiovascular diseases, miR-7 has been linked to coronary artery disease risk. Studies have shown that miR-7a/b overexpression enhances cardiac function by reducing cardiac remodeling, myocardial fibrosis, and cell apoptosis following MI. This is achieved by the direct binding of miR-7a/b to SP1, resulting in SP1 inactivation, thereby inhibiting PARP-1 and caspase-3 expression and exerting protective effects against cardiac remodeling and hypoxic injury [49]. Additionally, miR-7a/b demonstrates an anti-fibrotic effect by directly binding to SP1, reducing SP1 expression, and inhibiting cardiac fibroblast proliferation and migration, as well as the TGF beta and MAPK pathways, hence mitigating cardiac fibrosis [50].

### 4.3. Myocardial Hypertrophy and Cardiomyopathy

In response to pathological stress, the heart frequently undergoes abnormal remodeling and hypertrophic growth. Long-lasting cardiomyocyte hypertrophy indicates an increased risk of heart failure or even cardiac death. In recent years, several studies have highlighted the significant role of SP1 in cardiomyocyte growth and cardiac hypertrophy. Angiotensin II (AngII) present in myocardial tissue has been shown to elevate calcium ion concentration in the myocardial cytoplasm, stimulate vascular smooth muscle contraction, increase cardiac afterload, and contribute to myocardial hypertrophy. The study by Sun et al. indicated that inhibiting SP1/Gata4 activation effectively decreased ANP expression, thus suppressing the re-expression of cardiac hypertrophic traits associated with genes like ANP and BNP [51]. Meanwhile, the research by Azakie et al. demonstrated that cardiac hypertrophy in a clinically relevant sheep model resulted from an increase in left-to-right shunt, left ventricular preload, and right ventricular afterload, accompanied by elevated SP1 expression and reduced SP3 expression in all four chambers, aligning with SP1 activation and SP3 inhibition of cTnT promoter activity. The researchers hypothesized that SP1 might compete with SP3 for binding to the cTnT promoter, leading to promoter activation and subsequent mediation of the cyclic adenosine monophosphate (cAMP) pathway, increased calcium influx, and elevated cytosolic calcium concentration through a calcium-triggered release mechanism, ultimately inducing cardiomyocyte hypertrophy [52]. Furthermore, upregulation of SP1 was also observed in recent studies, where it interacted with KLF5, lncRNA CTBP1-AS2, SYHE1-AS1, PCDH17, SNHG14, and PED5 promoter regions to influence cardiac hypertrophy development [53,54,55,56,57]. Additionally, hypertrophic cardiomyopathy (HCM) is a prevalent inherited heart disease characterized by myocardial hypertrophy and interstitial fibrosis. Zhang et al.’s recent work found that cardiac-specific conditional knockout SP1 mice exhibited a typical HCM phenotype, showcasing significant cardiac hypertrophy, interstitial fibrosis, and myofilament disorganization. Mechanistic investigations revealed that SP1 deficiency led to reduced binding to the Tuft1 promoter region, significantly lowering Tuft1 expression and contributing to the development of HCM [58]. Cumulatively, these studies suggest that targeting SP1 could be a promising approach to managing pathological cardiac hypertrophy and HCM.

Diabetic cardiomyopathy emerges as a leading cause of mortality and morbidity in diabetic individuals, characterized by myocardial apoptosis and fibrosis with a multifaceted etiology. Studies have implicated SP1 in the progression of diabetic cardiomyopathy. Chen et al. identified inhibited miR-30c expression and abnormal elevation of autophagy levels in blood samples from diabetic cardiomyopathy patients. They further revealed SP1 as a critical transcription factor regulating energy metabolism and serving as a key activator upstream of miR-30c. The exacerbation of cardiac anomalies was attributed to autophagy escalation from diminished miR-30c expression and BECN1 activation [59]. MICU1, a pivotal regulator of mitochondrial Ca(2+) uptake, exerts significant control over mitochondrial oxidative phosphorylation and redox equilibrium. Ji et al. discovered that the upregulation of SP1 enhances the expression of MICU1, which suppresses cardiomyocyte apoptosis induced by high glucose and high fat by increasing mitochondrial Ca(2+) uptake and activating the antioxidant system [60]. Subsequently, Roman et al. observed that miR-181c was downregulated through SP1 oxidation and MICU1 in obese/type-2 diabetes, promoting reactive oxygen species (ROS) production and elevating mitochondrial calcium levels, resulting in cardiac injury [61]. Therefore, SP1 plays a significant role in the progression of diabetic cardiomyopathy by controlling cardiomyocyte apoptosis. Additionally, during diabetes, sustained endothelial injury leads to the trans-differentiation of endothelial cells into mesenchymal cells through endothelial–mesenchymal transition (EndMT), ultimately transforming their phenotype into myofibroblasts. EndMT is deemed a crucial mechanism in diabetic cardiac fibrosis. Du et al. found that the upregulation of SP1 activates KLK8 transcription, consequently increasing the expression of TGF-β1 and pro-EndMT target genes in the TGF-β1/Smad signaling pathway, ultimately leading to myocardial fibrosis [62].

### 4.4. Vascular Disorders

Vascular cell proliferation, apoptosis, and phenotypic transformation are pivotal factors in the onset and progression of vascular diseases. Vascular calcification serves as a critical link between cardiovascular disease and end-stage renal disease, aging, and diabetes and poses a significant risk for cardiovascular mortality. Mechanisms contributing to calcification in vascular smooth muscle cells (VSMCs) include apoptosis, the release of apoptotic metabolites, matrix vesicles (MVs), the phenotypic transition of VSMCs to osteoblast-like cells, and matrix remodeling. Studies indicate that SP1 directly stimulates the transcription of bone morphogenetic protein 2 (BMP2), fostering calcification in the F9 cell model. Thus, Zhang et al. speculated whether SP1 participates in vascular calcification. Their research involved inducing vascular calcification in male Wistar rats using nicotine and vitamin D3, then treating them with adenoviruses carrying SP1 knockdown or no-load. They discovered that SP1 binds to the BMP2 promoter region, activating BMP2 transcription, thereby promoting phenotypic transformation, apoptosis, and MV release of VSMCs, ultimately resulting in calcium deposition and vascular calcification [14]. In addition, it has been discovered by Li et al. that SP1 interacts with Setd8 to activate Mark4 transcription, leading to the promotion of vascular calcification through Akt-mediated anti-apoptotic effects. The microtubule affinity-regulating kinase 4 (Mark4) is a serine/threonine protein kinase known for its ability to trigger cell apoptosis and autophagy by modulating Akt phosphorylation [63]. Furthermore, vascular calcification implicates a range of proteins, cytokines, and transcription factors regulating bone homeostasis and mineralization. As vascular calcification and bone loss occur concurrently, Di Bartolo et al. hypothesized that factors like osteoprotegerin (OPG) and calcium-inhibiting osteoclastic bone resorption could prevent arterial calcification. In vitro studies on human-derived VSMCs revealed that calcium and OPG regulate IGF1R expression through SP1. IGF1R stands out as a potent anti-apoptotic factor in VSMCs, with increased expression inhibiting vascular calcification. In addition, a feedback mechanism is proposed where moderate calcium levels increase OPG through SP1 activation of IGF1R transcription, subsequently promoting VSMC survival and inhibiting calcium-induced calcification. Conversely, high calcium levels inhibit IGF1R expression and activity, leading to further vascular calcification [64].

Aortic aneurysm, a common vascular disease in Western countries affecting various parts of the aorta, is increasingly linked to vascular calcification in its development and progression [65]. The primary pathological mechanism involves the dedifferentiation of VSMCs into a synthetic phenotype, resulting in extracellular vesicle secretion, proliferation, and migration to repair damage, referred to as VSMC phenotypic transformation. Cyclooxygenase-2 (COX-2) is locally expressed in aortic aneurysms, catalyzing the synthesis of prostaglandin E2 (PGE2), which inhibits VSMC proliferation. Xu et al. established a hypoxia-induced human umbilical vein endothelial cell model showing that SP1 binds to the COX-2 promoter during oxygen deprivation, increasing PGE2 levels that inhibit VSMC proliferation and aortic aneurysm progression [66]. VSMC phenotypic transformation also contributes significantly to aortic dissection (AD) pathogenesis, with microRNAs playing crucial regulatory roles. Tang et al. identified miR-124 as a key regulator, showing decreased expression in clinical AD specimens and directly targeting the SP1 gene to regulate VSMC phenotypic transformation [67]. In addition, miR-335-5p has been involved in the pathogenesis of AD, and its mechanism includes the negative regulation of SP1 by miR-335-5p to suppress VSMC proliferation, migration, and phenotypic switching [68]. Thus, it is reasonable to conclude that SP1 mediates the regulation of VSMC phenotypic transformation.

Moreover, pulmonary arterial hypertension (PAH) is a chronic vascular disease characterized by cell proliferation and vascular remodeling, often leading to heart failure due to right ventricular overload. It has been found that reactive oxygen species (ROS) in pulmonary artery endothelial cells (HPAECs) of PAH patients produce NADPH oxidase 1 (NOX1), which, in turn, promotes the proliferation of endothelial cells (ECs) in vitro. By comparing lung tissue samples from PAH and non-PAH patients, Vallance et al. demonstrated that in PAH, NOX1-induced hypoxia activates SP1, promoting SP1 binding to the CXCL12 promoter and inducing CXCL12 transcription. This process leads to glutamine and glucose metabolism via CXCL12, promoting HPAEC proliferation and migration, implicating SP1 in regulating EC proliferation processes [69]. Furthermore, recent research has observed increased expression of Dickkopf 1 (DKK1) in HPAECs within both in vivo and in vitro PAH models. DKK1 promotes proliferation and inhibits apoptosis of human pulmonary arterial endothelial cells by maintaining redox homeostasis. Its mechanism depends on its interaction with serine hydroxymethyltransferase 2 (SHMT2) through the AKT-SP1 axis [70]. In general, SP1 is also involved in regulating the proliferation process of ECs.

### 4.5. Arrhythmia

Arrhythmia serves as the primary cause of cardiac arrest [71], with its pathological mechanism mainly involving inflammation, fibrosis, and autoimmune mechanisms by affecting either fibroblast activation-related electrical remodeling or the functionality of various cardiac ion channels [72]. Studies have indicated that SP1 plays a role in regulating the development of long QT syndrome (LQTS), ventricular arrhythmias, and atrial fibrillation (AF).

Within adult heart tissue, gap junction channels are crucial for transmitting action potentials from the sinoatrial node to the working myocardium. Among these channels, Connexin 40 (Cx40) is the most abundantly expressed in the mammalian atria and conduction system [73]. While somatic mutations have been suggested to potentially increase susceptibility to arrhythmias, the exact mechanism remains unclear. The presence of Cx40 mutations in atrial myocytes in the heart tissue of AF patients implies their significance in AF development. Research has shown that the core promoter region of Cx40 is co-regulated by NKX2-5, TBX5, GATA4, and SP1 [74]. Additionally, atrial fibrosis serves as a crucial marker of atrial remodeling in AF patients, characterized by abnormal proliferation of atrial fibroblasts and excessive extracellular matrix deposition. Long non-coding RNAs (lncRNAs) longer than 200 nt play a role in various biological processes, including cell growth, proliferation, migration, and myocardial fibrosis [75]. Studies have demonstrated increased expression of lncRNA plasmacytoma variant translocation 1 (PVT1) in atrial tissue and AngII-induced human atrial fibroblasts from AF patients, promoting atrial fibroblast proliferation and collagen production by competitively adsorbing miR-128-3p [76]. Therefore, SP1 is involved in the regulation of the development and progression of AF and may become a target for the treatment of AF in the future.

The rapid delayed rectifier potassium ion channel encoded by the human ether-á-go-go-related gene (hERG) is vital for phase 3 repolarization of the myocardial action potential, and mutations in the hERG gene lead to LQTS. SP1 has been identified as a counter-activator of the hERG gene, upregulating hERG expression and activating the potassium ion channel [77]. Furthermore, the role of SP1 in the transcription of the hERG gene has been highlighted. LQTS disturbs cardiac action potential repolarization and predisposes to ventricular arrhythmias such as ventricular fibrillation (VF), which is often fatal, and cardiac arrest. Many studies have found that the main cause of hERG channel obstruction is the hydrophobicity and aromaticity of Tyr 652 and Phe656, while SP1 transcriptional regulatory activity is largely determined by posttranslational modifications such as phosphorylation/dephosphorylation. Zhan et al. found that SP1 threonine (Thr)/tyrosine (Tyr) phosphorylation could be decreased by the PI3K/Akt pathway, thereby regulating the expression of the hERG channel [78]. Thus, SP1 acts primarily through the regulation of the hERG channel in LQTS and VF.

### 4.6. Others

Hypertensive heart disease is a significant contributor to cardiovascular morbidity and mortality. Among the many causes of hypertension, endothelial dysfunction is a common cause [79]. SP3, like SP1, belongs to the Sp/KLF family and is closely associated with cardiovascular development [80]. Studies have shown that endothelial cell-specific SP1/SP3 deficiency leads to endothelial dysfunction, primarily by regulating the expression of AMPKa [81]. Cardiac remodeling in hypertension is characterized by cardiac hypertrophy, fibrosis, and inflammation, potentially leading to left ventricular dysfunction and heart failure. While Smad7 is known for its negative regulatory role in various inflammatory conditions, its specific involvement in hypertensive diseases remains unclear. In a study conducted by Wei et al., an AngII-induced model of cardiac remodeling in hypertensive mice demonstrated a notable decrease in Smad7 expression in hypertensive heart tissue. Further investigations revealed that the Smad7 deletion led to SP1 activation. SP1 is crucial for AngII-induced myocardial fibrosis and inflammation, with cardiac fibrosis being mediated through the SP1/TGF-beta/Smad3 signaling pathway and inflammation via the SP1/NF-κB/miR-29 signaling pathway [82]. Thus, SP1 plays a vital regulatory role in hypertensive heart disease.

Moreover, SP1 also contributes to the regulation of sepsis-induced myocardial injury. Sepsis can result in multiple organ dysfunctions, with myocardial injury being a severe complication often leading to fatality. Studies have shown a close association between apoptosis, autophagy, pyroptosis, and septic cardiac dysfunction. Liu et al. identified a significant increase in ZFAS1 expression in a mouse model of sepsis and septic cardiomyocytes. Mechanistic insights unveiled that SP1 activated ZFAS1 expression, thereby mediating cardiomyocyte pyroptosis and autophagy through the miR-590-3p/AMPK/mTOR signaling pathway, ultimately exacerbating sepsis-induced cardiac dysfunction [83]. Additionally, Xu et al. delved into the intrinsic mechanism of miR-208a-5p in regulating sepsis-induced myocardial injury. Given the pivotal role of microRNAs in infectious myocardial injury, they discovered that SP1 enhances miR-208a transcription by binding to the miR-208a promoter region. Consequently, miR-208a-5p inhibits the expression of the X-linked inhibitor of apoptosis protein (XIAP), which is an important caspase inhibitor in the process of apoptosis, leading to myocardial apoptosis and further promoting sepsis-induced cardiac dysfunction [84]. However, miR-124, a member of the microRNA family, has been shown to alleviate sepsis-induced cardiomyocyte apoptosis. The research discovered that SP1 interacts with miR-124-3p to inhibit histone deacetylase 4 (HDAC4), a promising therapeutic target for sepsis. This interaction led to an enhancement in sepsis-induced myocardial injury, possibly related to the downregulation of hypoxia-inducing factor 1α (HIF-1α), which is the HDAC4 client transcription factor [85]. Therefore, SP1 interacts with members of the microRNA family, participating in the regulation of sepsis-induced myocardial damage, but regulating different microRNAs will have different effects. This intricate network of cellular processes sheds light on the underlying mechanisms of sepsis-induced cardiac dysfunction. The interplay between apoptosis, autophagy, and pyroptosis, orchestrated by key players such as ZFAS1 and miR-208a-5p, unravels a complex web of regulatory pathways that contribute to myocardial injury in the context of sepsis. Understanding these molecular interactions is crucial in developing targeted interventions to mitigate the impact of organ dysfunctions in critically ill patients.

## 5. Perspective and Prospective

**Figure 2:**
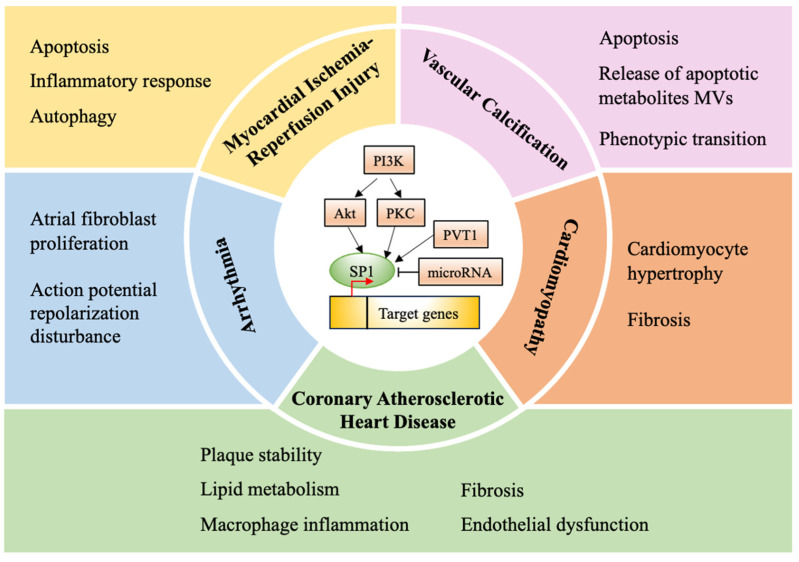
The role of SP1 in gene transcription regulation. SP1 can either stimulate or suppress gene transcription by binding to Guanine–Cytosine (GC)-rich regions within target gene promoters or through post-translational modifications such as phosphorylation. SP1 is crucial in controlling processes such as apoptosis, cell hypertrophy, autophagy, inflammation, lipid metabolism, plaque stabilization, endothelial dysfunction, fibrosis, and other pathological pathways. These processes significantly influence the development and progression of pathological conditions such as coronary atherosclerotic heart disease, ischemia-reperfusion injury, cardiomyopathy, arrhythmia, and vascular calcification. The straight arrows indicate the promotion of SP1 expression, while the T-shaped ends indicate inhibition. The red arrow represents target gene promoters.

Cardiac disease is the primary cause of death globally and is closely linked to high rates of morbidity and mortality. SP1 is a crucial transcription factor that regulates various cellular functions. It plays a significant role in cell growth, differentiation, apoptosis, ferroptosis, and carcinogenesis [11,86]. This review focuses on the involvement of SP1 in various cardiac diseases, including coronary heart disease, ischemia-reperfusion injury, cardiomyopathy, arrhythmia, and vascular disease (Figure 2). Understanding the regulation of SP1 could lead to improved treatment for these heart conditions. SP1 is implicated in regulating cardiac MIRI development by impacting apoptosis, inflammatory responses, and oxidative stress. The upregulation of SP1 can mitigate intracellular calcium overload and ease MIRI in cardiomyocytes by increasing the expression of MICU1 and SERCA2a. However, further investigation is needed to clarify the regulatory role of SP1 in MIRI inflammatory responses. Additionally, SP1 may offer protection against coronary atherosclerosis and cardiac remodeling post-myocardial infarction by influencing the gene transcription of PSRC1, ABCA1, SR-BI, and P4Ha1. SP1 regulates processes like inflammation, lipid metabolism, plaque stability, endothelial dysfunction, and fibrosis, thereby delaying the progression of cardiac remodeling. Moreover, SP1 contributes to cardiac hypertrophy through the activation of gene transcription, impacting diseases like pathological cardiac hypertrophy and HCM differently due to distinct pathogenesis. Furthermore, SP1 is involved in the regulation of diabetic cardiomyopathy and myocardial fibrosis by affecting autophagy, apoptosis, oxidative stress, and EndMT. Its role in vascular calcification remains unclear, but timely expression regulation of SP1 may prove effective in treating vascular calcification. Additionally, SP1 plays a role in clinical vascular diseases like aortic aneurysm, aortic dissection, and pulmonary hypertension. Lastly, SP1 influences arrhythmogenesis by affecting atrial fibroblast proliferation, collagen production, and hERG channels. It promotes AF by activating the TGF-beta1/Smad signaling pathway and counteracts the hERG gene, blocking LQTS progression, which can be fatal.

The Sp/KLF family participates in various growth-related signal transduction pathways, impacting cell proliferation positively or negatively [10]. Sp1 binding sites have been previously identified in enhancer/promoter regions of certain growth- and cell-cycle-regulated genes. Sp1 undergoes increasing phosphorylation during the G1 phase of the cell cycle [22]. Afify recognized SP1 as one of the key transcription factors involved in heart regeneration [87]. Various studies have delved into the role of SP1 in regulating neonatal cardiomyocyte proliferation, mainly by controlling the transcription of Fgf2 and Fgfr [88,89]. The fundamental mechanism through which SP1 governs cardiomyocyte proliferation and cardiac regeneration remains obscure and warrants further investigation. Furthermore, the involvement of SP1 in diseases like dilated cardiomyopathy and other arrhythmias should be validated through fundamental and clinical experiments. Exploring additional regulatory pathways for SP1-mediated apoptosis, myocardial fibrosis, vascular calcification, and other pathological processes, as well as identifying other target genes, is crucial for a comprehensive study.

Taken together, although certain aspects of SP1’s molecular actions remain enigmatic and demand validation through more in-depth research, these findings open new avenues for unraveling the significance of SP1 in cardiac disorders. They offer insights into disease mechanisms and hint at the potential for uncovering enhanced diagnostic, therapeutic, and prognostic indicators. Therefore, future research should focus on the following key areas to enhance our understanding of specificity protein 1 (SP1) in cardiac health: mechanism elucidation (how SP1 contributes to inflammation and myocardial ischemia-reperfusion injury to develop targeted treatments), genetic impact (key role of SP1 in gene regulation related to coronary atherosclerosis and cardiac remodeling to identify therapeutic targets), effects of SP1 in cardiac conditions (cardiac hypertrophy, cardiomyopathies, and vascular diseases), as well as arrhythmias and cardiac regeneration. We would then reveal SP1’s therapeutic potential and improve management strategies for cardiac disorders.
